# The Relationship Between Trait Procrastination, Internet Use, and Psychological Functioning: Results From a Community Sample of German Adolescents

**DOI:** 10.3389/fpsyg.2018.00913

**Published:** 2018-06-11

**Authors:** Leonard Reinecke, Adrian Meier, Manfred E. Beutel, Christian Schemer, Birgit Stark, Klaus Wölfling, Kai W. Müller

**Affiliations:** ^1^Department of Communication, Johannes Gutenberg University Mainz, Mainz, Germany; ^2^Outpatient Clinic for Behavioral Addictions, Department of Psychosomatic Medicine and Psychotherapy, University Medical Center, Johannes Gutenberg University Mainz, Mainz, Germany

**Keywords:** procrastination, Internet use, adolescents, self-control, mental health

## Abstract

Adolescents with a strong tendency for irrational task delay (i.e., high trait procrastination) may be particularly prone to use Internet applications simultaneously to other tasks (e.g., during homework) and in an insufficiently controlled fashion. Both Internet multitasking and insufficiently controlled Internet usage may thus amplify the negative mental health implications that have frequently been associated with trait procrastination. The present study explored this role of Internet multitasking and insufficiently controlled Internet use for the relationship between trait procrastination and impaired psychological functioning in a community sample of *N* = 818 early and middle adolescents. Results from multiple regression analyses indicate that trait procrastination was positively related to Internet multitasking and insufficiently controlled Internet use. Insufficiently controlled Internet use, but not Internet multitasking, was found to partially statistically mediate the association between trait procrastination and adolescents’ psychological functioning (i.e., stress, sleep quality, and relationship satisfaction with parents). The study underlines that adolescents with high levels of trait procrastination may have an increased risk for negative outcomes of insufficiently controlled Internet use.

## Introduction

Various lines of research suggest that certain uses of digital media and Internet applications (e.g., social media) are associated with impaired psychological functioning among adolescents (e.g., Tsitsika et al., 2014; van der Schuur et al., 2015; Müller et al., 2018). Specifically, a number of recent studies observed that Internet applications are frequently used as a means for dysfunctional procrastination (e.g., Lavoie and Pychyl, 2001; Meier et al., 2016). While the negative influence of procrastination on psychological functioning has been consistently documented in numerous studies (van Eerde, 2003; Steel, 2007; Sirois, 2016), the role of Internet use as an activity that contributes to the negative association between trait procrastination and mental health has only recently been demonstrated in a representative sample of adult Internet users (Reinecke et al., 2018). The present study aims to build on and extend this research by investigating, whether Internet multitasking and insufficiently controlled Internet use link trait procrastination to impaired psychological functioning among adolescents, a group of particularly avid Internet users (Lenhart, 2015).

Ubiquitously available options for entertaining Internet use and computer-mediated communication have a particularly high appeal for adolescents, as they afford high autonomy, opportunities for identity exploration, and a digital space that is often largely free of parental control (Jordan, 2018). However, this heightened digital autonomy comes at a price: adolescents are challenged to self-control their media usage to a previously unknown extent (Hefner et al., 2018), for instance, in self-directed learning environments (i.e., during homework). Seminal work on the delay of gratification among preschool children underlines the importance of self-control and procrastination among young individuals for their later success in life (Mischel et al., 1989). However, previous procrastination research has predominantly addressed procrastination based on adult or student samples (e.g., Tice and Baumeister, 1997), whereas only few studies have investigated procrastination among early and middle adolescents between the ages of 10 to 17 (e.g., Pychyl et al., 2002). Consequently, very little is known about the impact of procrastination on the psychological functioning of adolescents and the role Internet use may play in this context.

The present study attempts to contribute to existing research in the context of procrastination in three different ways: (1) By addressing the associations between trait procrastination and psychological functioning among adolescents—a particularly vulnerable yet understudied population of procrastinators. (2) By exploring the role of Internet use—a ubiquitous alternative activity constantly available to procrastinators—as a potential statistical mediator of the associations between trait procrastination and impaired psychological functioning. (3) By comparing the relevance of two different forms of Internet use (Internet multitasking and insufficiently controlled Internet use) in the context of procrastination, thus providing a theoretical bridge between the literature on procrastination and research addressing potentially problematic forms of Internet use.

In the following, we will thus first summarize existing research on the link between trait procrastination and psychological functioning, with a particular focus on adolescents. We then discuss how Internet use may statistically mediate this relationship as it is often used simultaneously to individuals’ primary tasks and particularly difficult to control. We then proceed to test the interplay between trait procrastination, Internet use, and psychological functioning in a community sample of German adolescents. Results are discussed with regard to implications for procrastination research and adolescents’ self-control in a constantly connected world.

### Trait Procrastination and Psychological Functioning in Adolescence

*Procrastination* is a quintessential self-control failure (Steel, 2007) and has been defined as “the voluntary delay of an intended and necessary and/or [personally] important activity, despite expecting potential negative consequences that outweigh the positive consequences of the delay” (Klingsieck, 2013, p. 23). In contrast to strategic or rational delay, procrastination is a term used for dysfunctional or irrational delay that is likely to result in more harm than good (at least in the long term). Building on this definition, *trait procrastination* refers to relatively stable interindividual differences in procrastinatory behavior across various life domains. Research clearly shows that such differences are partly rooted in individuals’ genetic makeup, specifically their impulsivity (Gustavson et al., 2014), and in major personality traits, particularly conscientiousness (Steel, 2007; Steel and Klingsieck, 2016).

A large body of literature has linked trait procrastination to impaired psychological functioning and reduced mental health (van Eerde, 2003; Steel, 2007; Sirois, 2016). A representative study of the German population aged 14 to 95, for instance, reports that trait procrastination was related to various indicators of impaired mental health, including stress, depression, anxiety, fatigue, and reduced life satisfaction (Beutel et al., 2016). While procrastination showed similar mean levels across the life span in this study, it was especially pronounced in the youngest age group (14–29-year-olds), warranting particular attention to this sub-population.

A prevalent negative mental health outcome of procrastination is *perceived stress*. In a longitudinal study among university students, for instance, Tice and Baumeister (1997) found that procrastination diminishes stress initially, but results in higher stress levels when the deadline of a task approaches. Naturally, stress arises when the procrastinators rush to complete their intended tasks on time (e.g., Lay and Schouwenburg, 1993). Hence, stress has often been found to mediate the relationship between procrastination and other (mental) health outcomes (Sirois et al., 2003, 2015; Stead et al., 2010; Sirois, 2016). Moreover, a small meta-analysis of four studies by Sirois and Kitner (2015) identified maladaptive coping as a central mechanism linking procrastination to stress experiences. Apparently, procrastinators are more prone to respond to everyday life stressors and task-specific challenges with avoidant or non-constructive coping. As stress-inducing states are not eliminated or mitigated, procrastinators experience higher levels of stress (Sirois and Kitner, 2015). Increased stress is thus a key outcome of trait procrastination.

Beyond stress, procrastination has been linked to impaired general health and, specifically, reduced *sleep quality*. Sirois et al. (2015) found higher levels of procrastination to be correlated with increased sleep latency, daytime dysfunction, and use of sleep medication as well as reduced sleep duration. Two processes, beyond heightened stress levels, may explain this link: first, procrastinators tend to report increased worry (Stöber and Joormann, 2001), rumination (Stainton et al., 2000), and guilt (Pychyl et al., 2000). Constant cognitive-affective preoccupation with one’s own daily dilatory behavior and its potential detrimental consequences may easily extend to the late evening hours and impede falling asleep. Second, emerging research finds bedtime procrastination, that is, “failing to go to bed at the intended time, while no external circumstances prevent a person from doing so” (Kroese et al., 2014, p. 1) to be a frequent problem for many individuals. Overall, this research points to a detrimental effect of procrastination on sleep quality.

Finally, there is some evidence hinting at negative effects of procrastination in the *interpersonal domain*. Frequent procrastination impairs performance in work and academic environments (Tice and Baumeister, 1997; Kim and Seo, 2015), which can result in lower salaries or even unemployment (Nguyen et al., 2013) and may thus compromise personal relationships with family, friends, fellow students, and coworkers. Ferrari et al. (1999) investigated the social support networks of procrastinators and indeed found that frequent procrastinators suffered from poorer family relations and experienced their interpersonal relationships as less satisfying, indicating a negative effect of procrastination-related interpersonal conflict. Among adolescents, such interpersonal troubles may become particularly salient in the family domain. Repeatedly procrastinating homework or exam preparations is likely to be noticed by parents eventually. This may spark homework-related conflict between adolescents and their parents (Dumont et al., 2012). Accordingly, if trait procrastination increases the likelihood of parent–child conflict about academic performance, it should be negatively associated with adolescents’ satisfaction with their interpersonal relationships to their parents.

In contrast to research on the link between trait procrastination and psychological functioning among (emerging) adults, research on the role of procrastination in adolescents’ lives is surprisingly scarce. Milgram et al. (1995) and Milgram and Toubiana (1999) as well as Ferrari and Olivette (1993, 1994) were among the first to investigate procrastination specifically in this population. They found that adolescents—similar to adults—tended to procrastinate more on unpleasant (rather than pleasant) academic tasks (Milgram et al., 1995) as well as on tasks that made them anxious (Milgram and Toubiana, 1999). Results by Owens and Newbegin (1997) also support the assumption that academic procrastination in school is negatively related to adolescents’ academic achievement. Several studies from the United States, Canada, and Singapore (Lay et al., 1998; Scher and Osterman, 2002; Klassen et al., 2009) further indicate that procrastination among school-aged children and adolescents is strongly related to conscientiousness and self-control capabilities. Together, these studies substantiate the notion that procrastination can be consistently explained as a phenomenon of self-control failure across age groups (Steel, 2007).

While abundant research shows a negative impact of trait procrastination on (mental) health and interpersonal functioning in the general population and particularly among university students, research investigating such effects among school-aged children and adolescents is lacking so far. Our synopsis of studies overall indicates that the nomological network of procrastination among young individuals does not deviate from that of older age groups. Nonetheless, the relationship between trait procrastination and mental health has not been investigated systematically in a large sample of adolescents. We aim to close this gap by testing whether the links between trait procrastination, perceived stress, sleep quality, and interpersonal relationships observed among adults also extend to adolescents:

(1) Trait procrastination will be positively related to perceived stress;
(1) Trait procrastination will be positively related to sleep problems;
(1) Trait procrastination will be negatively related to relationship satisfaction with parents.

### The Role of Internet Use for Procrastination and Psychological Functioning

Beyond testing the *direct* relationship between trait procrastination and psychological functioning among adolescents, the second major goal of the present study is to explore how trait procrastination and impaired psychological functioning are connected *indirectly* via the alternative activities that procrastinators pursue instead of their intended activity. Specifically, the present study addressed the role of Internet use as a potential link between trait procrastination and psychological functioning. A growing number of studies suggest that media in general (Panek, 2014; Reinecke et al., 2014; Reinecke and Hofmann, 2016) and Internet applications in particular (Lavoie and Pychyl, 2001; Hinsch and Sheldon, 2013; Meier et al., 2016) are frequently used for procrastination. Building on the definition of general procrastination (Klingsieck, 2013), we define *procrastinatory Internet use* as the voluntary delay of an intended and necessary and/or [personally] important activity *in preference of an Internet-enabled alternative activity*, despite expecting potential negative consequences that outweigh the positive consequences of the delay.

Little attention has been given to the unique role of such alternative activities in procrastination research so far. Survey participants and researchers have described these activities as pleasant and enjoyable as well as distracting or tempting (Pychyl et al., 2000; Sirois and Pychyl, 2013). One study found that susceptibility to fun activities such as meeting friends, watching TV, or online surfing predicted procrastination among college students (Dewitte and Schouwenburg, 2002). Social interaction, in particular, was found to be a tempting alternative activity for academic procrastinators (Schouwenburg and Groenewoud, 2001). We argue that Internet use is a particularly likely alternative activity for procrastinators for three reasons (Hofmann et al., 2017): (1) Thanks to mobile Internet connections and smartphones, media content and communication are almost ubiquitous and permanently available at virtually no cost or effort, making Internet use ‘top of mind’ almost constantly for many (young) individuals (Klimmt et al., 2018). (2) Furthermore, many online activities (e.g., social media use, gaming, and watching videos) promise instant gratifications and pleasurable experiences and should thus be particularly attractive to procrastinators seeking mood optimization when facing an aversive or difficult task (Sirois and Pychyl, 2013). (3) Finally, media use in general (LaRose, 2010) and Internet use in particular (Van Koningsbruggen et al., 2017) is often highly habitualized, making impulsive, insufficiently controlled selection and—as a consequence—procrastinatory use of Internet applications particularly likely (Meier et al., 2016; Schnauber et al., in press). In fact, the results of an experience sampling study by Reinecke and Hofmann (2016) demonstrate that more than 60% of media use episodes (i.e., TV, video game, or work-unrelated Internet use) conflicted with other goals such as “effective time use” or “not delaying things” (p. 452). This suggests a high prevalence of procrastinatory media use in everyday life. Further evidence supporting the notion that Internet applications are particularly frequently used for procrastination comes from a survey by Lavoie and Pychyl (2001): study participants perceived almost half of the total time spent online as procrastination. More recently, research has also investigated social media use as a procrastinatory activity. Two studies by Meier et al. (2016) demonstrate that Facebook is frequently used for procrastination among college students, and research by Hinsch and Sheldon (2013) reveals that reductions in overall social media use were associated with decreases in general procrastination.

Besides the high prevalence of Internet use as a procrastinatory activity, the extant literature also provides first evidence of negative associations between procrastinatory Internet use and psychological functioning. Two studies found procrastinatory Internet use to be associated with negative self-evaluative emotions such as guilt (Panek, 2014; Reinecke and Hofmann, 2016). Furthermore, procrastinatory Internet use has been linked to reduced life satisfaction (Hinsch and Sheldon, 2013) and subjective well-being (Reinecke and Hofmann, 2016) as well as increased levels of academic stress (Meier et al., 2016). Taken together, these findings suggest that various Internet uses seem to be among the most prevalent alternative activities procrastinators give in to and they may partly account for the negative associations between trait procrastination and psychological functioning. Preliminary evidence supporting this rationale is provided by findings from Reinecke et al. (2018). In a representative survey of the German population, the authors found a positive relationship between trait procrastination and insufficiently controlled Internet use. The resulting negative consequences of Internet use, in turn, partially mediated the negative effects of trait procrastination on mental health. These mechanisms should be especially relevant in adolescence, since teenagers and young adults are among the most avid Internet users (Lenhart, 2015). The second goal of the present study was thus to replicate and extend the findings of Reinecke et al. (2018) among adolescents.

### Trait Procrastination as a Predictor of Internet Multitasking and Insufficiently Controlled Internet Use

In a first step, we aimed at exploring whether and how trait procrastination influences the situational contexts in which adolescents use the Internet. More specifically, we were interested in the relationship between trait procrastination and Internet multitasking. The term *Internet multitasking* refers to “any combination of Internet use with other media or non-media activities” (Reinecke et al., 2017, p. 94) and thus describes situations in which the Internet is used concurrently or in short succession with other tasks or activities. Notably, Internet multitasking is not equivalent with procrastinatory Internet use. There may be instances of concurrent use of the Internet and other tasks that do not result in dysfunctional task delay (e.g., listening to an online music stream while doing homework). We suggest, however, that due to their increased tendency to seek pleasurable activities for short term mood optimization (Sirois and Pychyl, 2013), individuals with high levels of trait procrastination will use the Internet more often simultaneously to or in short succession with their primary tasks. According to this emotion regulation perspective (see also Pychyl and Sirois, 2016), procrastinators non-consciously seek short-term increases in positive affect in order to distract themselves from an aversive task. Increases in short-term affect and arousal also seem to be a common (expected) outcome of media multitasking (Wang and Tchernev, 2012; Yeykelis et al., 2014; Bachmann et al., 2018). Results of an experience sampling study by Bachmann et al. (2018), for instance, indicate that students’ multitasking with an autonomously selected additional activity was associated with increased positive affect (compared to mono-tasking). Among the most frequent multitasking activities were watching something/listening to music and social media use. As trait procrastinators are likely to show a stronger propensity for alternative activities that allow mood repair (Sirois and Pychyl, 2013) and since media multitasking seems to increase short-term positive affect, we expected trait procrastination to be a significant predictor of Internet multitasking:

(1) Trait procrastination will be positively related to Internet multitasking.

In addition to their tendency to engage in activities such as Internet multitasking that provide opportunities for mood repair, trait procrastinators may also show deficient levels of *self-control* over their Internet usage behavior. Trait procrastination has been consistently linked to lower levels of trait conscientiousness and self-control in meta-analyses (van Eerde, 2003; Steel, 2007) and to high impulsivity in genetic research (Gustavson et al., 2014). It thus appears reasonable to assume that trait procrastinators should have a higher risk of turning to alternative activities such as Internet use due to self-control failure. Accordingly, trait procrastinators may be more prone to use the Internet unintendedly or longer than planned. Previous research on so called ‘problematic Internet use’ supports this rationale.^1^ In a survey study by Thatcher et al. (2008), problematic Internet use was significantly positively related to using the Internet for procrastination. Some researchers even consider procrastination a central dimension or indicator of problematic Internet use (Davis et al., 2002). First empirical evidence for a significant relationship between trait procrastination and insufficiently controlled Internet use in the general population is provided by Reinecke et al. (2018). Consequently, we also expected to find a positive association between trait procrastination and insufficiently controlled Internet use in the sub-population of adolescents:

(1) Trait procrastination will be positively related to insufficiently controlled Internet use.

### Potential Associations Between Internet Use and Psychological Functioning

The tendency of trait procrastinators to engage in Internet multitasking and use the Internet in an insufficiently controlled fashion may have detrimental consequences. In fact, both research on media multitasking (van der Schuur et al., 2015) as well as literature addressing the impact of insufficiently controlled Internet use (Beutel et al., 2011; Müller et al., 2013) supports this rationale.

The results of a representative survey study by Reinecke et al. (2017) demonstrate that Internet multitasking was associated with increased levels of stress and impaired psychological health in the general population. In a systematic review of the extant literature on the effects of media multitasking on youths’ functioning, van der Schuur et al. (2015) conclude that multitasking is related to deficits in cognitive control (e.g., concentration), declines in academic performance, and negative consequences for emotional (e.g., depression and anxiety) as well as social functioning (e.g., social success).

Similar concerns like those associated with Internet multitasking have also been raised regarding the negative psychological consequences of insufficiently controlled Internet use. In a study by Müller et al. (2013), insufficiently controlled use was associated with a significant increase in negative consequences for social life, family, work, and health. Tsitsika et al. (2014) explored the psychological consequences of insufficiently controlled Internet use in a sample of over 13,000 adolescents from seven European countries and found a positive statistical relationship with a number of psychological symptoms. Insufficiently controlled Internet use has been associated with impaired mental health in other populations and age groups as well (Beutel et al., 2011; Müller et al., 2013, 2014). Beyond stress and psychological symptoms, prior research has also linked insufficiently controlled Internet use and its consequences to sleep problems. Research by Cheung and Wong (2011) demonstrates that adolescents showing problematic Internet use patterns also scored significantly lower on multiple dimensions of sleep quality compared to a control group of adolescents with unproblematic Internet use behavior. With regard to family functioning, a study by Choo et al. (2014) found a negative relationship between uncontrolled video-game use among children and adolescents and parent–child closeness. Similarly, in a study by Yen et al. (2007), problematic Internet use was linked to increased intra-family conflict and lower levels of family function.

Together, the extant research thus suggests that two specific behavioral patterns resulting from high levels of trait procrastination—Internet multitasking and insufficiently controlled Internet use—should directly increase adolescents’ risk for experiencing impaired psychological functioning:

(1) Internet multitasking is positively related to (a) stress as well as (b) sleep problems and negatively related to (c) relationship satisfaction with parents;
(1) Insufficiently controlled Internet use is positively related to (a) stress as well as (b) sleep problems and negatively related to (c) relationship satisfaction with parents.

As a consequence, and in conjunction with H3 and H4, we propose that the tendency of trait procrastinators to engage in Internet multitasking and to show insufficient control over their Internet use (see H2) should increase their risk for experiencing negative consequences resulting from Internet use. We thus suggest that Internet multitasking and insufficiently controlled Internet use will both partially statistically mediate the negative associations between trait procrastination and psychological functioning:

(1) Internet multitasking partially mediates the association of trait procrastination with (a) stress, (b) sleep problems, and (c) relationship satisfaction with parents;
(1) Insufficiently controlled Internet use partially mediates the association of trait procrastination with (a) stress, (b) sleep problems, and (c) relationship satisfaction with parents.

## Materials and Methods

### Participants and Procedure

All hypotheses were tested with data from a community-based probability sample of *N* = 818 adolescents from the federal state of Rhineland-Palatinate, Germany. The stratification was based on region, school type, and age and encompassed 14 sampling units (i.e., schools). The response rate of the schools contacted amounted to 68%. Before data collection, the adolescent participants as well as their parents were asked to provide written informed consent. Participation was voluntary, the study was approved by the Ethics Commission of the State Chamber of Medicine of Rhineland-Palatinate as well as the data protection commissioner of Rhineland-Palatinate and corresponded to the Declaration of Helsinki. Written and informed consent was obtained from the participants and the parents/legal guardians of all participants. Data collection took place in the classrooms and was supervised by an experienced member of the research team. Participants’ (53.8% female) age ranged between 10 and 19 years (*M* = 13.00, *SD* = 1.17). The majority of participants reported using the Internet daily (25.4%) or several times per day (46.0%), with an average daily usage time of *M* = 3.11 h (*SD* = 2.59).^2^

### Measures

#### Trait Procrastination

The validated German translation (Klingsieck and Fries, 2012) of the nine item short form of the General Procrastination Scale (Lay, 1986) was used to measure trait procrastination. Participants responded to the items (e.g., “I do not do assignments until just before they are to be handed in”) on a scale from 1 “very untypical” to 4 “very typical.” In the present study, the nine items showed a satisfactory internal consistency (Cronbach’s α = 0.87).

#### Perceived Stress

The four item short version (Warttig et al., 2013) of the Perceived Stress Scale (Cohen et al., 1983) was used to assess perceived stress. The four items (e.g., “In the last month how often have you felt difficulties were piling up so high that you could not overcome them?”) ask respondents to report how often they have experienced stress-inducing situations over the past month on a scale from 1 “never” to 5 “very often.” One of the items (“In the last month how often have you felt confident about your ability to handle your personal problems?”) significantly reduced the internal consistency of the scale and was thus removed for further analysis. The remaining three items showed an acceptable internal consistency (α = 0.64).

#### Sleep Problems

One item from the Patient Health Questionnaire (PHQ; Spitzer et al., 1999) was adapted to measure sleep problems. Participants responded to the item (“How often did you experience difficulties falling asleep over the last 2 weeks?”) on a scale from 0 “not at all” to 3 “almost every day” (*M* = 0.93, *SD* = 0.94).

#### Relationship Satisfaction With Parents

Three items adapted from the Network of Relationships Inventory (Furman and Buhrmester, 1985) were used to evaluate how satisfied the participants were with the relationship with their parents. Participants responded to the items (e.g., “How satisfied are you with your relationship with your parents?”) on a scale from 1 “not at all” to 5 “very much.” The scale showed a high internal consistency (α = 0.90).

#### Insufficiently Controlled Internet Use

Four items of the Assessment of Internet and Computer Game Addiction Scale (Müller et al., 2014) were used to measure insufficiently controlled Internet use. Participants responded to the items (e.g., “How often are you online although you had not intended to do so?”) on a scale from 0 “never” to 4 “very often.” The scale showed a satisfactory internal consistency (α = 0.72).

#### Internet Multitasking

Five items adapted from Reinecke et al. (2017) were used to assess Internet multitasking. Participants reported how often they use the Internet while they simultaneously (1) use other media, (2) are in a conversation with other people, (3) are having a meal, (4) go out with their friends, and (5) work on their homework on a scale from 1 “never” to 5 “very frequently.” The items showed a satisfactory internal consistency (α = 0.78).

## Results

All statistical analyses were performed with SPSS 23. The means, standard deviations, and zero-order correlations among all studied variables are presented in **Table 1**. Hypotheses 1–4 were tested using hierarchical regression analyses, controlling for the influence of three control variables: participants’ gender and age as well as general Internet use frequency (measured on a seven-point scale form 1 “never” to 7 “several times per day”). Detailed results of the regression analyses are presented in **Table 2**.

**Table 1:** Means, standard deviations, and zero-order correlations of study variables.

	*n*	*M*	*SD*	1	2	3	4	5	6	7	8	9
(1) Age	814	12.95	1,17	-								
(2) Gender (1 = male, 2 = female)	806	1.55	0,50	0.00	-							
(3) Internet use frequency	818	5.95	1,32	0.23**	-0.05	-						
(4) Trait procrastination	789	2.34	0,64	0.14**	0.00	0.31**	-					
(5) Stress	787	2.50	0,82	0.11**	0.25**	0.13**	0.34**	-				
(6) Sleep problems	640	0.94	0,95	0.13**	0.17**	0.18**	0.27**	0.32**	-			
(7) Relationship satisfaction with parents	658	4.31	0,86	-0.04	-0.05	-0.10*	-0.21**	-0.35**	-0.17**	-		
(8) Insufficiently controlled Internet use	774	1.24	0,75	0.04	0.08*	0.36**	0.52**	0.38**	0.27**	-0.21^∗∗^	–	
(9) Internet multitasking	765	2.38	0,85	0.17**	0.07	0.42**	0.43**	0.20**	0.20**	-0.13^∗∗^	0.45^∗∗^	-


*^∗^*p* < 0.05; ^∗∗^*p* < 0.01.*

**Table 2:** Summary of results of hierarchical regression analyses.

Predictor	Impaired control over internet use				Internet multitasking				Stress				Sleep problems				Relationship satisfaction with parents			
					
	*B*	*SE*	β	*p*	*B*	*SE*	B	*p*	*B*	*SE*	β	*p*	*B*	*SE*	β	*p*	*B*	*SE*	B	*p*
**Step 1: control variables**																				
Intercept	0.26	0.30		0.36	0.15	0.34		0.67	0.72	0.34		<0.05	-1.27	0.44		<0.01	4.92	0.42		<0.001
Age	-0.04	0.02	-0.07	0.06	0.04	0.03	0.05	0.17	0.06	0.03	0.08	<0.05	0.07	0.03	0.09	<0.05	-0.01	0.03	-0.01	0.75
Sex (1 = male, 2 = female)	0.14	0.05	0.09	<0.01	0.13	0.06	0.08	<0.05	0.38	0.06	0.24	<0.001	0.39	0.08	0.21	<0.001	-0.05	0.07	-0.03	0.48
Internet use frequency	0.22	0.02	0.40	<0.001	0.26	0.02	0.41	<0.001	0.08	0.02	0.13	<0.01	0.12	0.03	0.17	<0.001	-0.06	0.03	-0.10	<0.05
**Step 2: main predictor variables**																				
Intercept	-0.32	0.27		0.22	-0.29	0.31		0.36	0.33	0.32		0.30	-1.56	0.43		<0.001	5.17	0.42		<0.001
Age	0.06	0.02	-0.1	<0.01	0.04	0.02	0.06	0.09	0.06	0.02	0.09	<0.05	0.07	0.03	0.09	<0.05	-0.01	0.03	-0.01	0.77
Sex (1 = male, 2 = female)	0.11	0.05	0.08	<0.05	0.1	0.05	0.06	0.08	0.35	0.06	0.22	<0.001	0.36	0.07	0.19	<0.001	-0.04	0.07	-0.02	0.60
Internet use frequency	0.11	0.02	0.19	<0.001	0.16	0.02	0.26	<0.001		0.02	-0.02	0.59	0.03	0.03	0.04	0.35	-0.00	0.03	-0.00	0.93
Trait procrastination	0.05	0.04	0.40	<0.001	0.03	0.01	0.23	<0.001	0.26	0.05	0.21	<0.001	0.19	0.07	0.13	<0.01	-0.19	0.07	-0.14	<0.01
Insufficiently controlled internet use	-	-	-	-	0.25	0.04	0.22	<0.001	0.27	0.05	0.26	<0.001	0.24	0.06	0.19	<0.001	-0.14	0.06	-0.13	<0.05
Internet multitasking	0.17	0.03	0.20	<0.001	-	-	-	-	-0.02	0.04	-0.02	0.69	0.04	0.05	0.03	0.49	-0.02	0.05	-0.02	0.76

**Explained variance**																				
Step 1	*ΔR*^2^ = 0.15, *p* < 0.001				*ΔR*^2^ = 0.18, *p* < 0.001				*ΔR*^2^ = 0.08, *p* < 0.001				*ΔR*^2^ = 0.08, *p* < 0.001				*ΔR*^2^ = 0.01, *p* = 0.09			
Step 2	*ΔR*^2^ = 0.22, *p* < 0.001				*ΔR*^2^ = 0.13, *p* < 0.001				*ΔR*^2^ = 0.14, *p* < 0.001				*ΔR*^2^ = 0.08, *p* < 0.001				*ΔR*^2^ = 0.05, *p* < 0.001			
Total	*R*^2^ = 0.37, *p* < 0.001				*R*^2^ = 0.31, *p* < 0.001				*R*^2^ = 0.22, *p* < 0.001				*R*^2^ = 0.16, *p* < 0.001				*R*^2^ = 0.06, *p* < 0.001			


**N* varies between models depending on missing values of predictor and outcome variables.*

The first hypotheses addressed the relationship between trait procrastination and the three indicators of psychological functioning, perceived stress (H1a), sleep problems (H1b), and relationship satisfaction with parents (H1c). As predicted in H1a and H1b, respectively, trait procrastination was positively related to perceived stress (β = 0.21, *p* < 0.001) and sleep problems (β = 0.13, *p* < 0.01). Supporting H1c, the data further revealed a negative relationship between trait procrastination and relationship satisfaction with parents (β = -0.14, *p* < 0.01).

The second hypothesis addressed the relationships between trait procrastination, Internet multitasking (H2a) and insufficiently controlled Internet use (H2b). As predicted in H2a, trait procrastination was positively related to Internet multitasking (β = 0.23, *p* < 0.001). Furthermore, supporting H2b trait procrastination was also positively related to insufficiently controlled Internet use (β = 0.40, *p* < 0.001).

Hypotheses 3 and 4 addressed the relationship between Internet multitasking and insufficiently controlled Internet use, respectively, with the three indicators of psychological functioning. Contrary to the predictions of H3, Internet multitasking was neither related to perceived stress (β = -0.02, *p* = 0.69), nor to sleep problems (β = 0.03, *p* = 0.49), nor to relationship satisfaction with parents (β = -0.02, *p* = 0.76). Hypothesis 3 was thus not supported by the data. In contrast to H3, however, the findings did support H4. Insufficiently controlled Internet use was significantly positively related to perceived stress (β = 0.26, *p* < 0.001) as well as sleep problems (β = 0.19, *p* < 0.001) and showed a significant negative association with relationship satisfaction with parents (β = -0.13, *p* < 0.05).

The indirect statistical effects of trait procrastination on psychological functioning via Internet multitasking (H5) and insufficiently controlled Internet use (H6) were bootstrapped with 5000 samples with replacement using the maximum likelihood method and 95% biased-corrected confidence intervals (CIs) with the PROCESS macro for SPSS (version 2.16; Hayes, 2018). Detailed results of the mediation analyses are presented in **Table 3**. Contrary to expectations, Internet multitasking neither statistically mediated the effect of trait procrastination on perceived stress (*B* = -0.01, 95% CI = [-0.04, 0.03]), nor on sleep problems (*B* = 0.01, 95% CI = [-0.03, 0.06]), nor on relationship satisfaction with parents (*B* = -0.01, 95% CI = [-0.05, 0.03]). H5 was thus not supported by the data. However, the data did reveal a significant indirect effect of trait procrastination via insufficiently controlled Internet use on perceived stress (*B* = 0.15, 95% CI = [0.18, 0.36]), sleep problems (*B* = 0.24, 95% CI = [0.12, 0.36]) and relationship satisfaction with parents (*B* = -0.14, 95% CI = [-0.26, -0.03]), thus supporting H6.

**Table 3:** Parallel mediation analyses between trait procrastination (*X*), Internet multitasking (*M1*), insufficiently controlled Internet use (*M2*), and psychological functioning (*Y*).

Predictor	Stress (*N* = 686)			Sleep problems (*N* = 559)			Relationship satisfaction with parents (*N* = 572)		
			
	*B*	*SE*	*95% CI*	*B*	*SE*	*95% CI*	*B*	*SE*	*95% CI*
Intercept	0.22	0.33	[-0.42, 0.86]	**-1.56**	**0.43**	**[**-**2.40, -0.72]**	**5.17**	**0.41**	**[4.36, 5.99]**
**Covariates**									
Age	**0.06**	**0.02**	**[0.01, 0.11]**	**0.07**	**0.03**	**[0.01, 0.14]**	-0.01	0.03	[-0.07, 0.05]
Sex (1 = male, 2 = female)	**0.34**	**0.06**	**[0.24, 0.45]**	**0.36**	**0.07**	**[0.21, 0.51]**	-0.04	0.07	[-0.18, 0.10]
Internet use frequency	-0.01	0.02	[-0.06, 0.03]	0.03	0.03	[-0.03, 0.09]	0.00	0.03	[-0.06, 0.06]
**Direct effects**									
Trait procrastination	**0.26**	**0.05**	**[0.16, 0.36]**	**0.19**	**0.07**	**[0.05, 0.33]**	**-0.19**	**0.07**	**[-0.32, -0.06]**
Impaired control over Internet use	**0.27**	**0.05**	**[0.18, 0.36]**	**0.24**	**0.06**	**[0.12, 0.36]**	**-0.14**	**0.06**	**[-0.26, -0.03]**
Internet multitasking	-0.02	0.04	[-0.09, 0.06]	0.04	0.05	[-0.07, 0.14]	-0.02	0.05	[-0.11, 0.08]
**Indirect effects of trait procrastination**									
Via insufficiently controlled Internet use	**0.15**	**0.03**	**[0.10, 0.21]**	**0.13**	**0.04**	**[0.07, 0.21]**	**-0.08**	**0.03**	**[-0.14, -0.01]**
Via Internet multitasking	-0.01	0.02	[-0.04, 0.03]	0.01	0.02	[-0.03, 0.06]	-0.01	0.02	[-0.05, 0.03]

Explained variance	*R*^2^ = 0.22, *p*<0.001			*R*^2^ = 0.16, *p*<0.001			*R*^2^ = 0.06, *p*<0.001		


**N* varies between models depending on missing values of predictor and outcome variables. All significance levels are based on 95% confidence intervals (CIs) with lower and upper bounds reported in brackets. For indirect effects, 95% bias-corrected CIs were calculated based on 5000 bootstrap samples with replacement. Significant effects are highlighted in bold.*

## Discussion

The present study had two main goals: first, we attempted to extend procrastination research by exploring the negative implications of trait procrastination for psychological functioning in the under-researched population of adolescents. Second, we aimed at investigating the role of Internet multitasking and insufficiently controlled Internet use as a potential mediator of the detrimental effects of trait procrastination on (mental) health.

Findings concerning our first set of hypotheses (H1a–H1c) clearly underline the need for future research to address procrastination and the resulting implications for psychological functioning in adolescents more systematically. In the present study, trait procrastination was significantly associated with impaired psychological functioning of adolescents in multiple domains. Trait procrastination showed a significant relationship with increased levels of stress, replicating similar results found for procrastination in the general population (e.g., Beutel et al., 2016). Furthermore, trait procrastination was positively related to sleep problems. Given the major influence of sleep quality on other central aspects of psychological functioning in adolescents, such as school performance (Wolfson and Carskadon, 2003), this finding further underlines the detrimental potential of procrastination for the development of adolescents. The results also extend prior research by suggesting that the risks associated with procrastination in adolescents may interact and intensify each other: previous research has already demonstrated the negative effects of procrastination on academic performance (Owens and Newbegin, 1997; Kim and Seo, 2015). Our findings suggest that procrastination may not only be directly related to decreased school performance (e.g., via irrational delay of homework or test preparations) but also indirectly via impaired sleep quality. This remains speculative, however, and needs to be tested in future research. Finally, our results demonstrate that trait procrastination was linked to detrimental effects in an important domain of adolescents’ interpersonal relationships, that is, their family life. In the present study, adolescents high in trait procrastination showed an increased tendency to report lower levels of satisfaction with the relationship with their parents. Given the central role of positive and supportive parent–adolescent relationships as a major protective factor in adolescent development (Parker and Benson, 2004), these findings further underline the developmental drawbacks associated with trait procrastination in this life phase.

Overall, these findings strongly underline that a more systematic and detailed understanding of the role of procrastination in adolescence is a pressing task for future research. While the present study suggests that adolescents suffer from similar negative consequence of procrastination as emerging adults or the general population (van Eerde, 2003; Steel, 2007; Beutel et al., 2016), adolescence is a particularly vulnerable time that confronts young individuals with numerous developmental tasks (Roisman et al., 2004). In this crucial period, the detrimental effects of procrastination may pose a particularly high risk for this age group that could impair the successful transition into adulthood. The long-term effects of procrastination during adolescence on psychological functioning, career success, and social and romantic relationships should thus be addressed in longitudinal study designs. Furthermore, a number of questions pertaining to the specific characteristics of procrastination in adolescence remain unanswered. It is unclear, for example, whether the vulnerability to the detrimental effects of procrastination or the predictors of procrastinatory behavior systematically differ between adolescents and the general population. Comparative research that contrasts procrastination and its consequences in different age groups would thus significantly extend our understanding of the role of procrastination in different life phases.

The findings with regard to our second set of hypotheses (H2–H6) clearly underline that trait procrastination is related to specific patterns of Internet usage behavior (i.e., Internet multitasking and insufficiently controlled Internet use) with unique implications for adolescents’ psychological functioning. Moreover, the results characterize insufficiently controlled Internet use as a partial statistical mediator of the negative relationship between trait procrastination and psychological functioning. As proposed in H2, higher levels of trait procrastination were associated with a stronger tendency for Internet multitasking and a higher risk of insufficiently controlled Internet use. This suggests that trait procrastination may affect both the *context* of Internet use (i.e., more frequent use of the Internet in co-presence with other tasks or responsibilities) as well as the *volitional control* over individual usage patterns (i.e., using Internet applications unintendedly or longer than intended). Our findings with regard to H3 and H4 demonstrate that insufficiently controlled Internet use, but not Internet multitasking, was negatively related to psychological functioning. More crucially for the context of procrastination, our data further show that insufficiently controlled Internet use partially statistically mediated the relationship between trait procrastination and stress, sleep quality, and relationship satisfaction with parents (H6), whereas Internet multitasking did not (H5). The present study thus supports and replicates the results of previous research underlining the important role of media use in general (Reinecke et al., 2014; Reinecke and Hofmann, 2016) as well as Internet use in specific (Hinsch and Sheldon, 2013; Meier et al., 2016) as an attractive alternative activity for procrastinators.

Furthermore, our findings significantly extend prior research in several ways. First, the present study is among the first to systematically explore the role of Internet use for procrastination in a large sample of adolescents. Given the central role of Internet-enabled activities in the everyday lives of adolescents and emerging adults (Lenhart, 2015; Jordan, 2018), this age groups seems to be particularly relevant for research on Internet use and procrastination. Our findings clearly demonstrate that procrastination is significantly related to Internet multitasking and insufficiently controlled Internet use in this life phase. Furthermore, the present study extends our understanding of the psychological effects of procrastinatory media use. Previous research has demonstrated that procrastinatory media use has negative effects on self-related emotions (e.g., guilt) and situational well-being (e.g., Reinecke et al., 2014; Reinecke and Hofmann, 2016), as well as negative long-term effects on psychological health (e.g., depression and anxiety) and academic stress (Meier et al., 2016). The present study extends these findings by revealing additional associations of Internet usage patterns resulting from trait procrastination with impaired sleep quality and adolescents’ satisfaction with the relationship to their parents. Additionally, the pattern of results found in the present study provides a more nuanced picture of the role of Internet use as a potential mediator of the negative effects of procrastination. The fact that Internet multitasking, in contrast to insufficiently controlled Internet use, did not statistically mediate the relationship between procrastination and impaired psychological functioning suggests that not all forms of Internet use associated with trait procrastination are necessarily detrimental. Many forms of Internet multitasking may be completely unproblematic for an individuals’ situational goals and obligations. While some instances of Internet multitasking could represent strategic forms of delay aiming at recovery from strain (Reinecke and Hofmann, 2016), other forms of Internet multitasking (e.g., searching for relevant information on Google while working on a homework assignment) may even directly contribute to task completion. Our findings thus suggests that the increased risk of insufficiently controlled Internet use, rather than a mere increase of the co-occurrence of Internet use and other activities, makes the Internet usage patterns associated with higher levels of trait procrastination potentially detrimental for mental health.

### Limitations

While the present study offers several new findings regarding the underlying processes and consequences of procrastination in adolescents, a number of limitations have to be taken into account. A first limitation refers to the cross-sectional nature of our research design that does not allow for causal inferences. The direction of relationships found in our analyses thus remains unclear. Although all of our hypotheses were carefully derived from theory and previous research, alternative interpretations of the data may be similarly plausible.

This particularly applies to the relationship between procrastination, psychological functioning, and insufficiently controlled Internet use. In the present study, impaired functioning was conceptualized as a result of procrastination as well as insufficiently controlled Internet use based on previous research supporting this assumption (Tice and Baumeister, 1997; Müller et al., 2013; Beutel et al., 2016). Other research, in contrast, proposes a reverse order of effects, suggesting that impaired psychological functioning could also be a *driver* of procrastination and insufficiently controlled Internet use, instead of *resulting* from them. Supporting this notion, previous research reveals that problematic and excessive Internet use can be interpreted as a maladaptive strategy to cope with psychological distress or critical life events (Li et al., 2010; Müller et al., 2018). Similarly, previous procrastination research has discussed the negative affective states associated with impaired mental health (e.g., depression or anxiety) as a potential driver of procrastination (van Eerde, 2003; Steel, 2007). Future research should thus explore the direction of effects between procrastination, insufficiently controlled Internet use, and psychological functioning in cross-lagged panel designs.

A further limitation of the present study refers to the use of self-report measures. The validity of the measurement of variables such as trait procrastination, psychological functioning, or insufficiently controlled Internet use may have suffered from social desirability effects. Furthermore, the massive prevalence of Internet use in the everyday lives of adolescents and the high number of ‘micro-episodes’ of Internet use over the day (e.g., quickly checking the smartphone) increasingly complicate adequate self-reports of individuals’ usage patterns (Schneider et al., 2017). Future research in this area would thus benefit from *in situ* measures of Internet use (e.g., via experience sampling or unobtrusive usage tracking) to provide a more detailed and less biased view on the interplay between Internet use and procrastination.

An additional limitation of the present research refers to the lack of information concerning the specific activities that adolescents engaged in online. Previous research demonstrates that social media such as Facebook seem to be particularly frequently used for procrastination by young adults (Hinsch and Sheldon, 2013; Meier et al., 2016). Furthermore, Reinecke et al. (2018) found that trait procrastination was more broadly associated with increased use of entertaining online content (i.e., social media, online video, online games, etc.) in the general population. As discussed above, different forms of Internet use (e.g., Internet multitasking vs. insufficiently controlled Internet use) but also different forms of Internet content (e.g., games vs. information) may vary in their relevance as procrastinatory activities and the resulting risks for psychological functioning. Exploring when and why procrastinators turn to different Internet applications and contents, but also other, non-media-related activities, as a means to procrastinate, thus remains an important task for future research.

A last limitation refers to the variance explained by the variables in the present study. While trait procrastination and insufficiently controlled Internet use were significant predictors of all three indicators of psychological functioning (stress, sleep problems, and relationship satisfaction), the total amount of variance explained was limited (see R^2^s and ΔR^2^s in **Table 2**). Moreover, while we argued for a link between trait procrastination and Internet multitasking based on the observation that multitasking can be a means for emotion regulation, we did not explicitly measure emotion regulation in this study. Together, this suggests that future research needs to incorporate additional variables and processes—such as emotional misregulation (Pychyl and Sirois, 2016)—that may help to provide a more complete understanding of the interplay of procrastination, Internet use, and the psychological functioning of adolescents.

## Author Contributions

KM, KW, and MB supervised and coordinated the data collection and data cleaning. LR and AM performed the statistical analyses and manuscript preparation. All authors significantly contributed to the theoretical and methodological development of this research.

## Conflict of Interest Statement

The authors declare that the research was conducted in the absence of any commercial or financial relationships that could be construed as a potential conflict of interest.

## Footnotes

1 While a fast growing number of studies has addressed addictive and problematic Internet use over the last two decades (for an overview, see Müller et al., 2017), the literature lacks consensus regarding the classification and definition of these forms of use (Beutel et al., 2011). While the concept of Internet addiction is frequently used in clinical research and refers to pathological forms of Internet use resulting in serious negative effects in various life-domains (Müller et al., 2017), the term problematic Internet use is often used to refer to milder or emergent forms of excessive use that do not represent the full spectrum of diagnostic criteria of Internet addiction (Tokunaga, 2015). Despite their conceptual differences, a key similarity of both problematic Internet use and Internet addiction lies in the central role of impaired control over Internet use which is a defining component of both concepts (Davis et al., 2002; Shaw and Black, 2008). Since insufficiently controlled Internet use is of key interest for the present study, research from both research traditions is reviewed conjointly in the following sections.

2 The present study was part of an omnibus survey addressing a number of additional research questions related to adolescent development and Interne use that are reported elsewhere (e.g., Müller et al., 2018). Due to the considerable length of the questionnaire and time restrictions at the participating schools, not all participants were able to respond to all scales included in the questionnaire. Depending on the position of individual constructs within the questionnaire, this resulted in varying numbers of missing values for the variables considered in the present study (see **Table 1** for details). The number of valid cases thus varies between individual analyses.
